# Detection of *Trichomonas vaginalis* in Vaginal Swab Clinical Samples from Palestinian Women by Culture

**DOI:** 10.5402/2011/872358

**Published:** 2011-12-10

**Authors:** Yasmeen Houso, Mohammad A. Farraj, As'ad Ramlawi, Tamer Essawi

**Affiliations:** ^1^Master Program in Clinical Laboratory Science, Birzeit University, P.O. Box 14, Birzeit, Palestine; ^2^Central Public Health Laboratory, Palestinian Ministry of Health, Ramallah, West Bank, Palestine

## Abstract

*Background*. Trichomoniasis is a common sexually transmitted disease caused by *Trichomonas vaginalis*. It is a major health problem worldwide. The World Health Organization (WHO) has estimated that 180 million infections are acquired annually worldwide. *Methodology*. Vaginal swabs (1207) were cultured for *T. vaginalis* on Trichomonas Medium no. 2 (Oxoid) soon after specimen collection. The cultures were examined daily using a light microscope to detect the presence of *T. vaginalis*. *Results*. The prevalence of *T. vaginalis* was 13.6% (164/1207). The infection rate was the highest during pregnancy, 28.1%, and the lowest among women whose spouses use condoms, 8.6%. *Conclusions*. The culture method was used in this study to accurately determine the prevalence of this parasite in the West Bank, Palestine. The results of the study will eliminate ambiguities concerning trichomoniasis in this country and will contribute to better management and proper treatment.

## 1. Introduction

Trichomoniasis, caused by *Trichomonas vaginalis*, is the most common sexually transmitted disease worldwide. According to the World Health Organization (WHO), an estimated 170 million new infections occur annually throughout the world [1]. Data on the prevalence and incidence rates of this parasite remains limited in many developing countries including Palestine [2]. According to syndromic diagnosis, the number of patients with trichomoniasis in Palestine in 2003 was 103 females and 36 males per 100,000 [3].

*T. vaginalis* is associated with diverse pregnancy outcomes and may serve as a cofactor in human immunodeficiency virus (HIV) transmission [4]. *T. vaginalis *increases the risk of HIV transmission as seen among African-Americans in the United States [4, 5]. *T. vaginalis* causes a common genitourinary infection, which is frequently asymptomatic; about 10–50% of infections in women are asymptomatic [6]. In women, trichomoniasis is characterized by a yellow-green frothy vaginal discharge, vaginal odor, pain with sexual intercourse, pain during urination, and vulvovaginal soreness and itching [7]. Complications in pregnant women include postabortion infection, postcesarean infection, preterm birth, low birth weight infants, and preterm labor causing premature rupture of the membranes [7]. *T. vaginalis* infection has been suggested as a risk factor for developing cervical neoplasia [8]. Other complications caused by *T. vaginalis* include pelvic inflammatory disease and tubal infertility, vaginitis, and urethritis [9]. In males, it is usually found in the urethra, prostate or epididymis causing urethritis and prostatitis [10].

Traditionally, physicians diagnose trichomoniasis on the basis of the clinical picture. Symptoms alone are not sufficient to make reliable diagnosis of* T. vaginalis* infection. Laboratory tests such as wet mount are routinely performed for this purpose. Although rapid and inexpensive, this technique has a low sensitivity of 50 to 80% [11–13]. For accurate diagnosis by wet mount, adequate numbers of the parasite must be present and the characteristic tumbling motility must be observed by an experienced technician within a short time [10].

The culture method was used in this study because it is the “gold standard” against which the performance of other diagnostic methods is compared [11, 12]. The aim of this study was to determine the prevalence of *T. vaginalis *among Palestinian women in the West Bank, Palestine. This is the first study conducted in this country that utilized the culture method and did not rely on direct microscopic examination.

## 2. Materials and Methods

### 2.1. Study Design and Target Population

This prospective study was conducted on women attending reproductive health clinics in the West Bank, Palestine. Unduplicated vaginal swabs were randomly collected by the attending physician from women residing in districts throughout the West Bank, Palestine. Informed consent was obtained from all women participating in the study.

### 2.2. Sample Collection and Processing

Samples were collected from 1207 women of childbearing age from October 2004 to December 2005. Vaginal discharge was taken from the posterior cervix or from the vaginal wall using a sterile swab containing Stuart's transport media (Via Magent 77/6, Rho (MI) Italy). The swabs were immediately transported to the Central Public Health Laboratory in Ramallah, Palestine and cultured within 4 hours after collection. Litmus paper was used to determine the pH of the discharge; the color change was compared with a pH standard color chart [14]. In most cases, a high vaginal pH (>4.5) is indicative of trichomoniasis.

The swabs were removed from the transport media and used to inoculate Trichomonas Medium no. 2 (Oxoid). The cultures were then incubated at 37°C and checked daily for motility under the microscope for a total of 5 days. 

### 2.3. Statistical Analysis

Chi-square was used to analyze the data obtained in this study at 0.05 level of significance using SPSS 13 statistical package (SPSS Inc., Chicago, Ill).

## 3. Results

This study was conducted on a total of 1207 women of childbearing age attending reproductive health clinics in the West Bank, Palestine. The culture results revealed a prevalence of 13.6% (164/1207). The rate of positive cultures for *T. vaginalis *was 22.7% (5/22) in women of 18 years and younger and 6.8% (4/59) in women of 46 years and older. Several variables were taken into consideration during this study. These variables included age, marital status, pregnancy and lactation, use of contraceptives, socioeconomic conditions, education, and symptoms. These results are summarized in Table 1.

The symptoms experienced by all women participating in this study are documented and summarized in Table 2. The percentage of positive cultures for *T. vaginalis* ranged from 13.1% (130/990) for vaginal discharge to 15.9% (48/302) for pelvic pain as shown in Table 2.

## 4. Discussion

*T. vaginalis *is the third most common cause of vaginitis. According to the WHO, *T. vaginalis *accounts for approximately half of all curable sexually transmitted diseases worldwide [1]. Annually it affects 170 million women worldwide [1]. The objective of this study was to determine the prevalence of trichomoniasis among Palestinian women by using the culture method.

Vaginal trichomoniasis is a recognized sexually transmitted disease. Sexually active women are at an increased risk of vaginitis due to this parasite. In this study, the prevalence of *T. vaginalis* was 13.6%. The prevalence of this parasite in Israel is 8.1% in patients with vaginitis [15]. Prevalence rates vary with different patient population and diagnostic methods. In this study, the percentage of positive cultures may correlate with sexual activity. As shown in Table 1, the percentage of positive cultures of *T. vaginalis* tends to decrease with advanced age as compared to young sexually active women. Although the number of women above 46 years of age was low (4/59), the low percentage of positive cultures of 6.8% may correlate with reduced sexual activity.

Interestingly, a study was conducted in Gaza (Palestine) using the Papanicolaou smears (Pap) to determine the prevalence of *T. vaginalis* among pregnant women [2]. The prevalence was 18.2% as compared to 28.1% obtained in this study. The Pap smears are not reliable and inaccurate for the diagnosis of *T. vaginalis* and have a low sensitivity of 61% [16] as compared to culture, the golden standard with a sensitivity of 80–95% and specificity exceeding 95%.

Considering women status, trichomoniasis is unusual in lactating women, since the glycogen-poor atrophic vagina of the breastfeeding woman is relatively hostile to this parasite [17]. Comparing the percentage of positive cultures found for married women (13.6%), pregnant women (28.1%), and lactating women (11.4%), *χ* ^2^ results were significant (*P* < 0.05).

The use of contraceptives is effective in reducing the percentage of positive cultures. In this study, the results obtained did not reveal a statistical significance (*P* > 0.05) in the percentage of positive cultures by using different means of contraceptives. The percentage was low (8.6%) in women whose spouses used condoms as compared to the others. This indicates that using barrier contraceptives (condoms) is the most effective mechanical barrier to various microorganisms [18]. Women using either barrier or oral contraceptives six months before becoming pregnant had decreased rates to be colonized by *T. vaginalis* as previously described [19].

Diagnosis of trichomoniasis is usually based on the presence of symptoms such as vaginal discharge, irritation, genital soreness, pelvic pain, burning sensation, and vaginal pH. Although 93% of the women had a vaginal pH > 4.5 (1125/1207) and 82% have vaginal discharge (990/1207), the percentage of positive cultures was 13.7% (154/1125) and 13.1% (130/990), respectively, as shown in Table 2. *T. vaginalis* grows best at acidic environment and attaches itself by its axostyle thus creating the irritation, inflammation, and other symptoms associated with the infection by this parasite [17]. There were no significant differences in the percentage of positive cultures of *T. vaginalis* according to occupation, education, or economic status.

In conclusion, this study showed that the prevalence of *T. vaginalis* in the West Bank is 13.6%. This relatively high number of positive cultures is of public health concern. This study warrants having better understanding and more accurate evaluation of urogenital trichomoniasis in Palestine. To curb the high rates of trichomoniasis and help in its prevention and control among Palestinian people, it is recommended that the Ministry of Health must take drastic measures to implement effective screening programs, provide treatment for infected partners, and spread awareness in the population about this disease and its transmission. In addition, it is extremely important to screen and treat asymptomatic women who may have persistent trichomoniasis for years.

## Figures and Tables

**Table 1:** The frequency of positive cultures of *T. vaginalis* in 1207 women according to different variables.

Variable		Positive cultures		*χ* ^2^
	Number of cases	Number	Percent	*P* value
Age groups (years)				
15–18	22	5	22.7	0.393
19–25	261	41	15.7	
26–30	255	35	13.7	
31–35	259	31	12	
36–45	307	43	14	
Over 46	59	4	6.8	

Totals	**1163**	**159**	**13.7**	

Marital status				
Married	1197	163	13.6	>0.05
Divorced	3	0	0	
Widow	7	1	14.3	

Totals	**1207**	**164**		

Women status				
Pregnant	178	50	28.9	<0.05
Lactating	324	37	11.4	
Other	705	77	10.9	

Total	**1207**	**164**	**13.6**	

Women contraceptive use				
IUD	458	53	11.6	0.791
Pills	130	12	9.2	
Others (coitus interrupts)	25	3	12.0	
Non-users	594	96	16.2	

Totals	**1207**	**164**	**13.6**	

Condom use				
Condoms used	81	7	8.6	<0.05
Condoms not used	1126	157	13.9	

Totals	**1207**	**164**	**13.6**	

Occupation				
Working women	59	6	10.2	0.463
Housewives	1130	155	13.7	

Totals	**1189**	**161**	**13.5**	

Education				
High school	1084	150	13.8	0.451
College	123	14	11.4	

Totals	**1207**	**164**	**13.6**	

Socioeconomic class				
Low	542	78	14.4	0.462
Moderate	646	83	12.8	
High	19	3	15.8	

Totals	**1207**	**164**	**13.6**	

Notes: married women have a stable sexual partner. All pregnant women are married. Socioeconomic classes are determined based on the poverty line put by the Palestine Central Bureau of Statistics, family income, the level of education, and profession.

**Table 2:** Positive cultures of *T. vaginalis* according to symptoms. Total number of samples is 1207.

Symptoms	Rate of symptoms		Positive for *T. vaginalis *	
	Number	%	Number	%
pH > 4	1125	93.2	154	13.7
Vaginal discharge	990	82	130	13.1
Urinary symptoms	489	40.5	67	13.7
Irritation	458	38	68	14.9
Soreness	382	31.7	57	14.9
Pelvic pain	302	25	48	15.9
